# Swallowing safety in Parkinson's disease after zona incerta deep brain stimulation

**DOI:** 10.1002/brb3.709

**Published:** 2017-04-21

**Authors:** Stina Sundstedt, Lina Holmén, Elin Rova, Jan Linder, Erik Nordh, Katarina Olofsson

**Affiliations:** ^1^ Division of OtorhinolaryngologyDepartment of Clinical SciencesUmeå UniversityUmeåSweden; ^2^ Division of Speech and Language PathologyDepartment of Clinical SciencesUmeå UniversityUmeåSweden; ^3^ Division of NeurologyDepartment of Pharmacology and Clinical NeurosciencesUmeå UniversityUmeåSweden; ^4^ Division NeurophysiologyDepartment of Pharmacology and Clinical NeurosciencesUmeå UniversityUmeåSweden

**Keywords:** body mass index, caudal zona incerta, deep brain stimulation, Parkinson's disease, swallowing function

## Abstract

**Objectives:**

The objective of this study was to examine swallowing function in patients with Parkinson's disease before and after caudal zona incerta deep brain (cZI DBS) surgery. The aims were to examine the effect of cZI DBS on swallowing safety regarding liquid and solid food, as well as to identify the effect of cZI DBS on body mass index (BMI) and specific items from part II of the Unified Parkinson's Disease Rating Scale (UPDRS).

**Materials and Methods:**

The median age of the 14 patients was 57 years (range 46–71), with a median disease duration of 6 years (range 2–13). The present sample is an extension of a previous report, into which six additional patients have been added. Fiber endoscopic examinations of swallowing function, measures of BMI, and evaluation of UPDRS part II items were made before and 12 months after surgery, with and without activated DBS.

**Results:**

There were no significant changes due to cZI DBS regarding penetration/aspiration, pharyngeal residue or premature spillage (*p *>* *.05). Median BMI increased by +1.1 kg/m^2^ 12 months after surgery (*p *=* *.01, *r *=* *.50). All reported specific symptoms from the UPDRS part II were slight or mild. A significant improvement regarding handling of utensils was seen 12 months postoperatively (*p *=* *.03, *r *= −.42).

**Conclusions:**

Caudal zona incerta DBS was found not to have a negative impact on swallowing safety. A significant increase in postoperative weight was observed, and speech seemed to be slightly negatively affected, whereas handling of utensils was improved with cZI DBS.

## 1 Introduction

Parkinson's disease (PD) is one of the most common neurodegenerative diseases in Europe (von Campenhausen et al., 2005), but the specific cause of the disease remains unknown (Kalia & Lang, 2015; von Campenhausen et al., 2005). The progression of PD is related to a successive neurodegeneration in substantia nigra, primarily by a loss of dopamine containing neurons (Gao & Hong, 2011; Kalia & Lang, 2015). The cardinal symptoms are resting tremor, rigidity, bradykinesia, and postural instability (Jankovic, 2008; Olanow, Stern, & Sethi, 2009). In addition, a range of secondary motor and non‐motor features are present, such as constipation, pronounced alterations in body weight, dysarthria, hypophonia, dystonia, sialorrhea, and dysphagia (Jankovic, 2008; Kalia & Lang, 2015; Olanow et al., 2009). Among the secondary motor features, dysphagia has the most serious consequences, as it may result in pneumonia secondary to aspiration. Dysphagia is also related to increased mortality rates (Fernandez & Lapane, 2002).

At present there is no cure for PD, but symptomatic treatment with dopaminergic drugs (L‐dopa) and with deep brain stimulation (DBS) alleviates the motor symptoms and improves quality of life (Deuschl et al., 2006; Olanow et al., 2009). In DBS treatment, the subthalamic nucleus (STN) is currently the most established target, but the posterior subthalamic area, including the caudal zona incerta (cZI), has been suggested as a superior target in selected cases (Plaha, Ben‐Shlomo, Patel, & Gill, 2006). Results from cZI DBS show similar or better results than from STN DBS, in terms of motor improvement as estimated by observer ratings of limb motor capacity (Burrows et al., 2012; Plaha et al., 2006). While such positive effects on limb movement are well documented in STN DBS, as well as in cZI DBS, the effects on swallowing function have not yet been conclusively evaluated (Troche, Brandimore, Foote, & Okun, 2013).

Disturbances in swallowing function may affect the oral, pharyngeal or esophageal phases (Dodds, Stewart, & Logemann, 1990). Dysphagic problems in PD may occur in all of these phases, with a prevalence of 82% (confidence interval 77%–87%; Kalf, de Swart, Ensink, & Bloem, 2008; Nilsson, Ekberg, Olsson, & Hindfelt, 1996). Compared to healthy controls, patients with PD show more post‐swallow pooling, as well as more silent saliva aspiration (Ali et al., 1996; Rodrigues, Nóbrega, Sampaio, Argolo, & Melo, 2011). Swallowing problems have also in general been associated with weight changes, and have been hypothesized to contribute to sialorrhea (Chou, Evatt, Hinson, & Kompoliti, 2007; Noziako, Saito, Matsumura, Miyai, & Kang, 1999). Sialorrhea has been reported in as many as 70%–78% of patients with PD (Chou et al., 2007), but neither the pathogenesis of sialorrhea in PD, nor the relationship between sialorrhea, L‐dopa treatment and DBS, have been fully elucidated (Chou et al., 2007). So far, there are no reports of sialorrhea or changes in body weight in patients treated with cZI DBS.

Several studies have investigated possible adverse effects after STN DBS. In a meta‐analysis by Kleiner‐Fisman et al. (2006), sialorrhea and weight gain were reported. Macia et al. (2004) and Barichella et al. (2003), who exclusively examined weight gain, reported increased weight in 100% of the patients, and listed several possible explanations for this. Of the suggested causes, they noted that STN DBS could decrease the patients’ energy expenditure (Kistner, Lhommée, & Krack, 2014), and that STN DBS could increase the sensitivity to food reward cues, hence increasing the inclination for eating (Serranová et al., 2011). No reports of the effect of cZI DBS on patients’ body weight are available.

With regard to swallowing function, neither L‐dopa treatment nor STN DBS seem to have a clinically significant effect on swallowing safety (Kulneff et al., 2013; Menezes & Melo, 2009; Silbergleit et al., 2012; Troche et al., 2013). Only one retrospective study has reported a significant deterioration of swallowing safety at 6 months after STN DBS (Troche et al., 2014). On the other hand, a number of studies, reviewed by Troche et al. (2013), showed no clear‐cut impact on swallowing parameters after STN DBS. Our previous report is the only available cZI DBS study that have evaluated the possible effects of cZI DBS on swallowing function (Sundstedt et al., 2012). The conclusion from that study was that cZI DBS did not have a negative impact on swallowing function in the eight patients that were included. The main limitations in our earlier report are the low number of participants and the high number of comparisons.

This study was performed to increase the power and strengthen the conclusions from the previous report while broadening the perspective by including a wider view on the swallowing. The purpose of this study was to examine swallowing function before and after cZI DBS surgery, with and without stimulation turned on. The aims were to examine the effect of cZI DBS treatment on swallowing safety with regard to liquid and solid food; to identify the effect of cZI DBS on the patients’ body mass index (BMI), as well as on the items “cutting food”, “sialorrhea”, “swallowing”, and “speech” as defined in the “Unified Parkinson's Disease Rating Scale” (UPDRS); and to study possible associations between BMI and swallowing function, as well as the selected UPDRS scores.

## 2 Material and Methods

**Figure 1:**
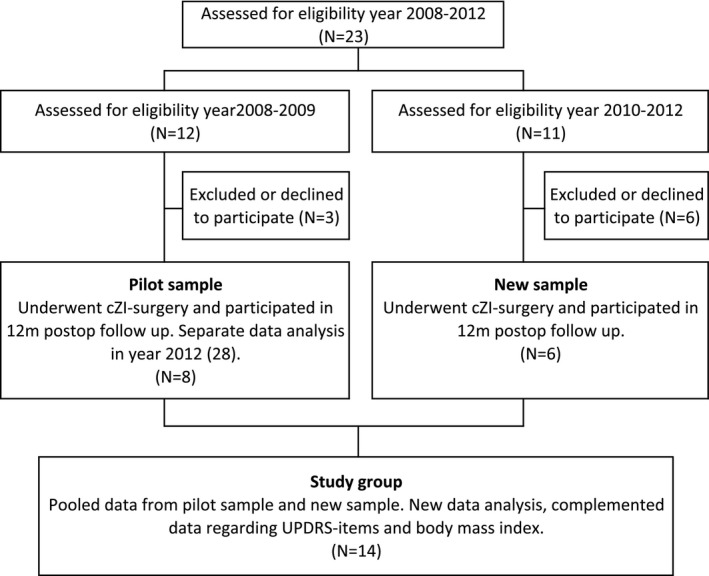
Description of samples included in the study

This prospective longitudinal study complements and extends our previous report on swallowing function (Sundstedt et al., 2012). Consecutive data from six new patients selected for cZI DBS were added to those from the eight patients included in an earlier pilot study (Sundstedt et al., 2012). The increase in number of participants contributes to a higher statistical power, which is the most important improvement in this extended study. Patients were evaluated for their suitability for inclusion in the study according to clinical evaluation and best clinical practice. Selection for bilateral cZI DBS surgery was based on overall motor function rating without regard to swallowing function. A total of 23 patients were screened for inclusion in the study, and 14 of them were included in the study group (Figure 1). Nine patients were excluded from the study; five of them failed the neuropsychological assessments, two were excluded because of unilateral DBS, one due to complications, and one patient declined to participate in the postoperative follow‐up. A detailed account of the patient characteristics is provided in Table 1. All subjects were given written information on the details of the study and gave their informed consent to participate according to the Helsinki declaration. The study design has been approved by the Regional Ethical Review Board in Umeå (08‐0934M).

**Table 1:** Patient characteristics at baseline

	Pilot sample^a^ (n = 8)	New sample (*n* = 6)	Pooled sample (*n* = 14)
	Median (range)	Median (range)	Median (range)
Age (years)	62 (49–71)	52 (46–66)	57 (46–71)
Disease duration (years)	6 (2–13)	8 (4–13)	6 (2–13)
Gender female/male (*n*/*n*)	2/6	0/6	2/12
BMI (kg/m^2^)	26.1 (18.4–30.9)	25.0 (23.0–38.4)	25.4 (18.4–38.4)
UPDRS‐III off med	36 (29–58)	41 (18–53)^b^	40 (18–58)^b^
UPDRS‐III on med	20 (10–42)	23 (8–45)^b^	23 (8–45)^b^
Hoehn and Yahr scale	2.5 (1.5–2.5)	2 (2–2)^c^	2.5 (1.5–2.5)^c^
LEDD (mg)	1,013 (300–1,997)	1,271 (0–2,412)	1,049 (0–2,412)
Anticholinergic medication	None	1 patient	1 patient
Indication for surgery	5 tremor, 2 on‐off fluctuations, 1 rigidity/bradykinesia	4 tremor +/‐ wearing off, 2 on‐off fluctuations	9 tremor +/‐ wearing off, 4 on‐off fluctuations, 1 rigidity/bradykinesia

BMI, body mass index; UPDRS‐III, motor part of Unified Parkinson's Disease Rating Scale, lower scores for better function. LEDD, levodopa daily equivalent dose.

a Patients included in pilot study by Sundstedt et al. (2012).

b Data missing from one patient.

c Data missing from three patients.

The assessment of the cZI target and the surgical procedure have been previously described in detail (Blomstedt, Sandvik, & Tisch, 2010). Pre‐ and post‐operative CT‐scans ensured that the active electrode location was within the cZI. The time frame of the study was from baseline to 12 months after cZI DBS. All evaluations were carried out with ongoing L‐dopa medication; and at the preoperative evaluation a dose that was 1.5 times the ordinary dose of L‐dopa equivalents was given. Postoperative evaluations were performed with “stimulation on” and “stimulation off”, with a 60‐min adaptation time after changes in the stimulator settings. Stimulation frequencies ranged between 125 and 160 Hz for all patients.

At the postoperative examinations, the PD medication was optimized for maximal effect on overall motor symptoms, and the evaluations were conducted within the optimal time frame of the patients’ usual medication cycle.

### 2.1 Evaluation of swallowing parameters, BMI and UPDRS items

Swallowing function was evaluated using fiber optic endoscopic evaluation of swallowing (FEES). The examinations were conducted using an Olympus ENF P4 transnasal flexible endoscope and a Wolf endocam 5502. In later examinations, an Olympus ENF VH flexible video endoscope combined with an Olympus CV ‐170 light source system were used.

The patients underwent a FEES examination, using one solid and four liquid consistencies. From these, the solid consistency, a biscuit covered with a smear of green jelly, and the thinnest liquid consistency, green dyed water, were analyzed in this study. These two were the last two consistencies to be swallowed during the examination. The biscuit and the water were always offered to the patient in a similar manner and the size of the bolus was a bite/gulp of freely chosen volume. The liquid and solid consistencies were chosen as they were the hardest to swallow and thus were hypothesized to best reveal potential swallowing problems. In the previous report all five consistencies were included in the analysis.

The video materials for the six additional patients were anonymized and randomized according to the same procedure as in our previous study (Sundstedt et al., 2012). For one of the previously analyzed patients, a minor part of a video recording was not available, and the missing scores were substituted by rating scale scores from the research notes made during the FEES examination. The videos were assessed together by the authors LH and ER. To ensure inter‐rater reliability, the author SS assessed 19% of the new material and authors LH and ER assessed 19% of the old material. Intra‐rater reliability was ensured by a second assessment of 17% of the swallowing examinations. Authors LH, ER, and SS were at the time of the rating speech language therapist in the last phase of training. Before the ratings were performed a few specific training sessions were carried out to ensure that the raters interpreted the rating scales in a similar manner.

The assessment protocol in this study comprised the Penetration/aspiration scale (0–7p; Rosenbek, Robbins, Roecker, Coyle, & Wood, 1996), the Secretion severity scale (0–3p; Murray, Langmore, Ginsberg, & Dostie, 1996), and evaluation of premature spillage (0–1p) and pharyngeal residual (0–1p). For all parameters, a lower score implies better function.

Patients’ weight and height, measured in the afternoon at the neurological ward at the occasion of the swallowing evaluations, were used for calculations of BMI (kg/m^2^).

Data regarding any occurrence of sialorrhea, speech or swallowing problems, or difficulties with handling utensils, were extracted from the UPDRS evaluations pre‐ and postoperatively. The assessments of the specific UPDRS items were administered by an experienced DBS‐nurse, during the patients’ stay on the neurologic ward. The scores were based on the patients’ experiences with their preoperative medication, and postoperatively with “medication on/stimulation on” at 12 months follow‐up. Scores from UPDRS range between 0 and 4p with the following labels: 0 = normal; 1 = slight problems; 2 = mild problems; 3 = moderate problems; 4 = severe problems. UPDRS scores were missing from one patient at the 12 months follow‐up.

### 2.2 Statistical analysis

All analyses were performed using SPSS version 20.0 for Mac. Descriptive statistics were provided as medians with ranges. Nonparametric two‐tailed tests were used and the significance level was set at 5%. Friedman repeated measures test by ranks was performed to test differences between conditions over time. Wilcoxon signed rank test was used for pairwise post hoc testing. Spearman's rho (*r*_s_) was used to test correlations between measures and for tests of intra‐ and inter‐rater reliability.

Estimated effect size was calculated according to the formula *r* = *z*/√*N*, where *N* is the number of observations, for example, *N*_observations_ = *n*_preop_ + *n*_postop_. This method yields standardized effect levels regardless of sample size and complements standard significance testing (Fritz, Morris, & Richler, 2012). Thresholds for qualitative descriptors of effect size were small (*r *>* *.10), moderate (*r *>* *.30), large (*r *>* *.50), and very large effect size (*r *>* *.70).

## 3 Results

### 3.1 Swallowing parameters, BMI and UPDRS items

**Figure 2:**
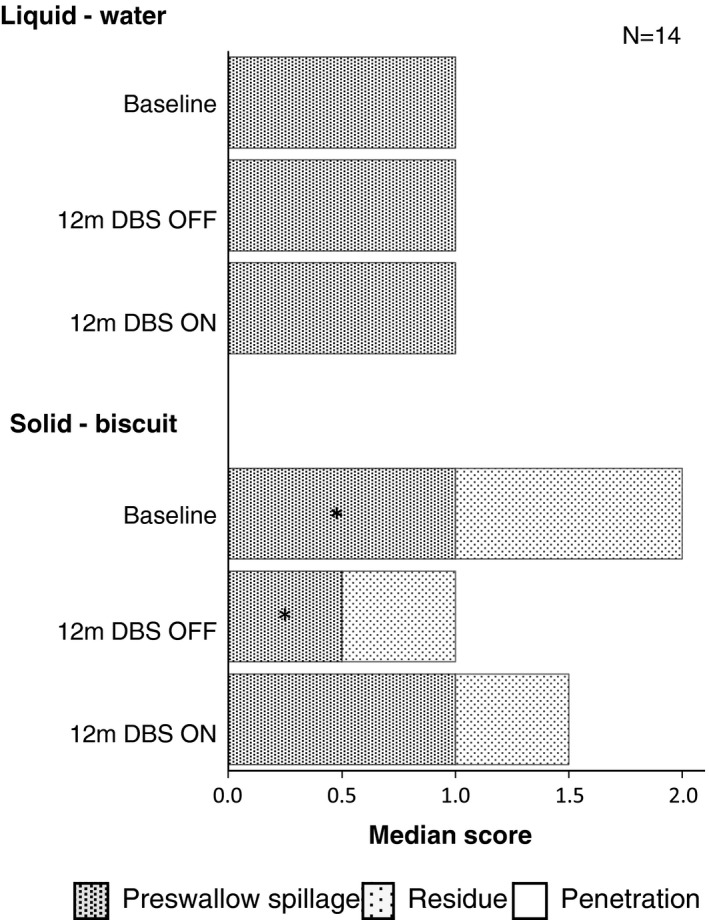
Median swallowing scores from fiber optic endoscopic evaluation of swallowing examinations. The lower the score the better the function. The median score for penetration/ aspiration was 0 for all conditions. *Preswallow spillage was significantly less at 12 m deep brain stimulation (DBS) OFF compared to baseline, *z *=* *−2.45, *p *=* *.01, *r *=* *.48. No other significant differences, *p *>* *.05

Descriptive statistics for swallowing parameters from FEES, BMI measurements and UPDRS part II scores are summarized in Table 2. Figure 2 illustrates the presence or absence of penetration/aspiration, pharyngeal residue, and premature spillage. There were no significant changes as a result of cZI DBS regarding penetration/aspiration, pharyngeal residue or premature spillage (*p *>* *.05).

**Table 2:** Medians, ranges and statistical tests for swallowing parameters, BMI and UPDRS items

Median (Range)	Baseline	12m Postoperative		Friedman test (*n* = 14)		
	Test dose of L‐dopa	Stim off	Stim on	*Fr*	*P*	
Liquid—Water						
Penetration/aspiration	0.0 (0–4)	0.0 (0–4)	0.0 (0–2)	3.00	.22	
Pharyngeal residue	0.0 (0–1)	0.0 (0–1)	0.0 (0–1)	0.25	.88	
Premature spillage	1.0 (0–1)	1.0 (0–1)	1.0 (0–1)	0.33	.85	
Solid—Biscuit						
Penetration/aspiration	0.0 (0–2)	0.0 (0–2)	0.0 (0–5)	1.2	.55	
Pharyngeal residue	1.0 (0–1)	0.5 (0–1)	0.5 (0–1)	0.33	*.85*	
Premature spillage	1.0 (0–1)*	0.5 (0–1)*	1.0 (0–1)	7.75	**.02**	
Secretion severity scale	0.5 (0–2)	0.0 (0–3)	0.0 (0–3)	0.00	1.00	
				Wilcoxon test		Effect size
				*z*	*P*	*r*
BMI (kg/m^2^) (*n* = 14)	25.4 (18.4–38.4)	—	26.7 (19.8–38.34)	2.67	**.01**	.50
UPDRS (0 − 4p) (*n* = 13)						
Cutting food	1.0 (0–2)	—	0.0 (0–1)	−2.13	**.03**	−.42
Sialorrhea	0.5 (0–2)	—	1.0 (0–2)	1.41	.16	.28
Swallowing	1.0 (0–2)	—	0.0 (0–2)	−0.38	.71	−.07
Speech	1.0 (0–2)	—	2.0 (1–2)	2.46	**.01**	.48

The lower the swallowing and Unified Parkinson's Disease Rating Scale (UPDRS) scores the better the function. Body mass index (BMI) >25.0 is regarded as overweight. Figures marked in bold text show significant differences. Friedman test statistics (Fr), p‐value (p), Wilcoxon test statistics (z) and estimated effect size *r *= *z*/√(*n*_preop_ + *n*_postop_).

*Significant according to Wilcoxon post‐hoc test *z *=* *−2.45, *p *=* *.01, *r *=* *−.48. *r *= *z*/√(*n*_preop_ + *n*_postop_).

**Figure 3:**
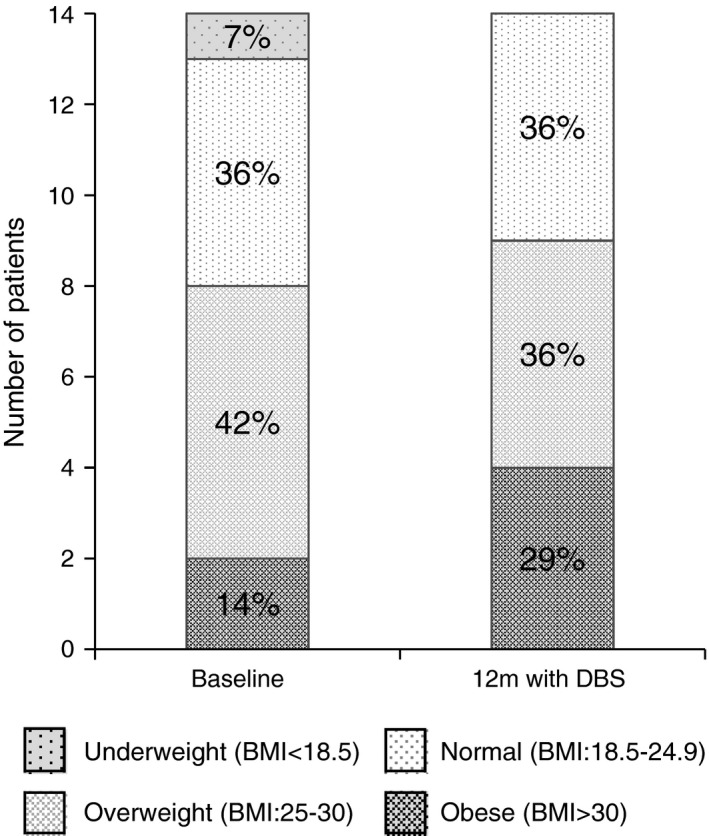
Shift in distribution of body mass index (BMI) 12 months after caudal zona incerta deep brain stimulation surgery. *N* = 14

The distribution of the 14 patients into different BMI categories at baseline and at 12 months after cZI DBS is illustrated in Figure 3. Comparison between baseline and 12 months postoperatively showed that one patient changed from underweight to normal weight, one patient changed from normal to overweight, while two others changed from overweight to obese. Ten patients were within the same BMI category at baseline and 12 months postoperatively. The median change in BMI was +1.1 kg/m^2^ (range ‐0.7 to 4.8). The median weight change in kg between baseline and 12 months follow‐up was +3.0 kg (range ‐3 to 15). Three patients lost 2–3 kg, five patients gained 1–4 kg, five patients gained 7–15 kg, and one patient stayed at the same weight pre‐ and postoperatively.

**Figure 4:**
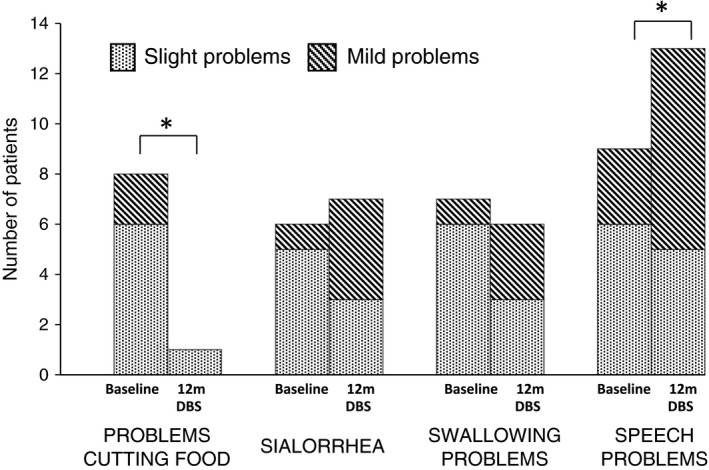
Number of patients with specific secondary motor and non‐motor symptoms according to Unified Parkinson's Disease Rating Scale, at baseline and 12 months after caudal zona incerta deep brain stimulation surgery. *N* = 13. *Significant differences according to repeated pair‐wise testing (see Table 2)

Prevalence of secondary and non‐motor features from UPDRS is seen in Figure 4. All reported symptoms were slight or mild, as no symptoms of severe or moderate character were observed. At baseline, eight of 13 patients had difficulties handling cutlery and cutting food with a knife. At 12 months, the patients managed cutlery significantly better, and only one of 13 patients reported difficulties. At baseline, 70% of the patients (9 of 13) had slight or mild problems with their speech, compared to 100% at 12 months after cZI DBS surgery.

### 3.2 Correlations between BMI, swallowing parameters and UPDRS items

At baseline, there were no significant correlations between BMI and any of the swallowing parameters from the FEES (*p *>* *.05). Neither were there any significant correlations between BMI and the items included from UPDRS (speech, cutting food, sialorrhea or swallowing, *p *>* *.05).

At the end point of the study, 12 months after cZI DBS surgery, a lower BMI was correlated with more preswallow spillage of liquids, as well as with more speech problems (DBS on, *r*_s_ = −.61, *p *=* *.02, and *r*_s_ = −.68, *p *=* *.01, respectively). BMI was not correlated with any of the other parameters from FEES or UPDRS (*p *>* *.05).

### 3.3 Inter‐rater and intra‐rater reliability

Tests of intra‐rater correlations with Spearman's rho for penetration/aspiration, premature spillage, pharyngeal residue and secretion severity scale showed high correlations (Pilot sample: *r*_s_ = .85, *r*_s_ = .81, *r*_s_ = .93, and *r*_s_ = 1.00, *p *<* *.01; Sundstedt et al., 2012). New sample: *r*_s_ = .89, *r*_s_ = .94, *r*_s_ = .79, and *r*_s_ = 1.00, *p *<* *.01). Tests of inter‐rater correlations for penetration, premature spillage, pharyngeal residue, and secretion severity scale showed moderate correlations (*r*_s_ = .68, *r*_s_ = .84, *r*_s_ = .57, and *r*_s_ = .63, *p *<* *.01).

## 4 Discussion

The purpose of the present report was to examine how different aspects of swallowing and eating are affected by cZI DBS, and it complements and extends our previous pilot study on swallowing function by including more patients, improving the analyzes regarding time points and consistencies as well as including data regarding BMI and UPDRS items (Sundstedt et al., 2012). The results from the FEES examination at baseline and 12 months after surgery indicate that cZi DBS does not have a negative influence on swallowing safety. At the 12 months postoperative evaluations, the occurrence of pharyngeal residue, and the penetration/aspiration of liquid or solid food were at unchanged levels compared to baseline, both with stimulation on and stimulation off. Compared to the preoperative baseline, the amount of premature spillage of solid and liquid food was the same as with cZI DBS turned on 12 months after surgery, while the amount of premature spillage of solid food was slightly reduced with DBS turned off. The results support our previously published data concluding that cZI DBS does not have a negative effect on swallowing (Sundstedt et al., 2012).

Deep brain stimulation in the posterior subthalamic area, including the cZI have earlier been found to alleviate general motor symptoms in PD (Plaha et al., 2006). The reason for the absent effect on swallowing function has not been clarified scientifically but might be due to that axial muscles are controlled in other ways and thus may respond differently to treatment, as compared to global motor extremities.

This study supports the notion that weight and BMI of patients with PD is increased at 12 months after cZI DBS surgery, with a large estimated effect size (*r *=* *.50), indicating that cZI DBS affects their BMI in a similar way to STN DBS. The median increase of BMI in our sample was 1.1 kg/m^2^, while the mean increase after STN DBS has been reported to range between 0.4 and 4.9 kg/m^2^ (Kistner et al., 2014). The results from the current study are unique as this is the only available study examining weight change in PD patients with cZI BDS. Further studies examining weight gain in cZI DBS and also studies comparing weight changes in patients with STN DBS and cZI DBS are needed to improve the preoperative information to patients.

The results from the specific UPDRS items suggest that approximately 50% of the patients in this study had slight or mild problems with sialorrhea and swallowing at baseline. However, cZI DBS does not seem to further affect the extent of sialorrhea or swallowing problems.

In this study, the ability to cut food improved significantly after cZI DBS surgery. The estimated effect size of this difference regarding cutting was moderate (*r *=* *.42). At 12 months after cZI DBS, one of 13 patients reported problems with handling utensils, as compared to baseline where eight of 13 stated problems with the handling of cutlery. This improvement is consistent with the expected general improvement of global motor function that is associated with DBS.

Minor speech problems were common at baseline (9 of 13 patients) and speech seems to be negatively affected by cZI DBS, in that all 13 patients reported problems with speech 12 months after cZI DBS surgery. The difference was significant and the estimated effect size was moderate (*r *=* *.48). A similar notion was made by Johansson et al. (2014) who have analyzed speech intelligibility in a parallel study based on partly the same sample as the current study. They concluded that speech intelligibility was negatively affected by cZI DBS. Regarding our study, it is, however, important to note that all problems were of a slight or mild character, and also that the UPDRS is not optimal for evaluation of speech.

At the 12 months postoperative evaluations, there was a correlation between lower BMI and impaired speech function, as well as between lower BMI and more premature spillage of liquid food. This could be interpreted as an indication of reduced motor control of the muscles involved in speaking and handling of food in the mouth, hence influencing food intake and indirectly body weight. Another reason for the association could be a natural progression of the disease, which also affects oral motor control and changes the metabolism.

In studies of possible effects of DBS at specific implantation sites on secondary motor tasks such as swallowing, the sample sizes are often small. A consequence of this may be studies with low statistical power, which can only detect large effects and risk disregarding moderate and small effects. When studying swallowing, it is important to detect moderate and small functional effects, as such disturbances may have a high impact on morbidity and mortality. Consequently, special attention should be given to sample sizes and statistical power in such studies. When statistically evaluating putative negative effects of treatment, it is furthermore important to avoid type II errors (Woods et al., 2006). In small samples, statistical tests may fail to find significant effects as a result of low power, while there is actually a negative impact. The number of patients in our previous study was small, because of a limited number of available patients (Sundstedt et al., 2012).

In the analyses of the current extended study, both significance testing and estimated effect size were used, to reinforce the capacity for detection of non‐significant differences with moderate or large effect sizes. Bonferroni corrections, sometimes used to counterbalance the effect of multiple significance testing on small samples, may in these situations further increase the risk of type II errors, and hence were not used in the present analysis (Nakagawa, 2004).

When the results from this study are interpreted it is important to remember that most patients had mild swallowing problems at baseline. The inclusion to the cZI surgery was based on patients’ overall motor function without regard to the swallowing function. The results can thus not be generalized to patients with PD that show substantial swallowing problems prior to DBS surgery.

A limitation of this study is that different raters performed the assessments of the swallowing data from the pilot sample and new sample (Sundstedt et al., 2012). However, the same raters always rated the same patients pre‐ and postoperatively. The raters were blinded to the patients’ status, time‐point, DBS status and swallowing function.

The strength of the study is its prospective longitudinal design with pre‐ and postoperative examinations and a specific examination protocol that was used for all examinations. This design makes it possible to detect effects on swallowing safety as a result of cZI DBS. Another strength was that inter‐rater and intra‐rater reliability were carefully investigated and found to be good.

## 5 Conclusion

The present extended study improves our knowledge of the effects on swallowing of DBS in general and DBS in the cZI in particular, which is of clinical importance as knowledge about swallowing function and weight changes after cZI DBS is very limited. The most important finding is that cZI DBS does not seem to have a negative impact on swallowing safety. Another important finding is that cZI DBS surgery may cause weight gain postoperatively. Furthermore, speech seemed to be slightly negatively affected, while handling of utensils was improved with cZI DBS.

## Conflicts of Interest

The authors report no conflicts of interest.
